# Tranexamic acid can reduce blood loss in adolescent scoliosis surgery: a systematic review and meta-analysis

**DOI:** 10.1186/s12891-023-06811-1

**Published:** 2023-08-29

**Authors:** Keyu Chen, Liang Wang, Qingyang Gao, Umar Masood, Zhimou Zeng, Huiliang Yang, Yueming Song

**Affiliations:** 1 Department of Orthopedic Surgery and Orthopedic Research Institute, West China Hospital, Sichuan University, Chengdu, 610041 China; 2 Jacobs School of Medicine and Biomedical Sciences, University at Buffalo, The State University of New York, Buffalo, NY USA

**Keywords:** Tranexamic acid, Adolescent scoliosis, Blood loss, Blood transfusion

## Abstract

**Background:**

Tranexamic acid (TXA) has been widely used in orthopedic surgery, but its efficacy in adolescent scoliosis (AS) surgery remains unclear in the literature. The purpose of this systematic review and meta-analysis is to evaluate the safety and efficacy of TXA compared to placebo treatment during or after AS surgery, by gathering data from randomized both controlled trials (RCTs) and non-RCTs.

**Methods:**

English and Chinese electronic databases including PubMed, Web of Science, Embase, Cochrane, CNKI, and Wan Fang database were searched to identify the relevant literature up until August 2022. The primary outcomes were intraoperative blood loss and total blood loss. The secondary outcomes included the need for transfusion, postoperative hemoglobin (Hb) level, and change in Hb level. Stata 17 was used for data analysis and the risk of bias was assessed. We followed the PRISMA checklist to ensure the quality of this article.

**Results:**

Twelve studies (795 participants) were included in the meta-analysis for intraoperative blood loss during surgery. The results suggest that TXA can reduce the intraoperative blood loss of the patients (MD = -306.40ml, 95%CI = -404.04ml to -208.77ml, p < 0.001). Six studies (2027 patients) were included in the meta-analysis for total blood loss. The pooled result shows that the total blood loss of the TXA group was significantly lower than that of the control group (MD = -779.24ml, 95% CI = -1157.10ml to -410.39ml, p < 0.001). Five studies (419 patients) were included in the meta-analysis for postoperative Hb level and shows a non-significant outcome (MD = 5.09 g/l, 95%CI = 2.92 g/l to 7.25 g/l, p = 0.611). Three studies (268 patients) were included in the meta-analysis for the postoperative Hb level. There is a non-significant decrease in the TXA group (MD = -0.23 g/l, 95%CI = -0.48 g/l to 0.01 g/l, p = 0.319). Eight studies (670 patients) reported data on the need for transfusion after surgery. The overall relative risks (RR) showed a significant difference between the TXA and control group, with a lower risk of transfusion in the TXA group (RR = 0.547, 95%CI = 0.308 to 0.972, p = 0.04).

**Conclusions:**

The meta-analysis of the data reveals that TXA usage is associated with a significant reduction in intraoperative and total blood loss, a lower risk of transfusion, and a non-significant change in postoperative Hb levels in AS surgery However, it should be noted that the surgical operation situations varied across different studies. Therefore, further research is required to investigate the effects of TXA on specific subgroups of gender, operation time, and blood transfusion indicators. Overall, our study provides valuable evidence for the clinical management of AS surgery and may inform the development of practice guidelines and protocols for the use of TXA in this setting.

**Supplementary Information:**

The online version contains supplementary material available at 10.1186/s12891-023-06811-1.

## Introduction

Scoliosis is a common adolescent condition that usually requires surgical correction. Posterior surgery is a common surgical strategy. In the posterior surgery, the spine is fixed through a system of fixation rods that have different crossover structures to avoid spinal movement after surgery, by cutting through the midline of the trunk and one side of the iliac spine. However, surgical correction for patients with adolescent scoliosis (AS) leads to large blood loss, commonly between 600 and 1500mL, because of the extensive cut bone and soft tissue dissection [1–5].

Thus, blood transfusions are often needed due to blood loss and low total blood volume. However, such transfusion sometimes leads to various sequelae, such as viral infections, allergic reactions, and fluid overload [6]. Therefore, hemostatics are applied during surgery to reduce blood loss. Tranexamic acid (TXA) is a synthetic antifibrinolytic agent acting at the lysine binding site of plasminogen, which inhibits plasminogen activation to plasmin, stops binding to fibrin, and inhibits fibrinolysis. The use of TXA has been shown to reduce bleeding during scoliosis surgery, thus minimizing the risks associated with transfusions [2, 7, 8].

Many studies have reported the important hemostatic effect of TXA on various operations, including scoliosis surgeries [8–11]. Yuan et al. [5] reviewed the publications before 2017 and found that TXA can decrease total blood loss and intraoperative blood loss during scoliosis surgery. High-dose TXA was found to be more effective than low-dose TXA in controlling blood loss. Shrestha et al. [2] reviewed 5 clinical trials and showed that a dosage of 100 mg/kg TXA is safe for AS patients. Researchers found that adolescents had less blood loss than adults due to fewer medical comorbidities and fewer rigid curves, which makes the prediction of blood loss easier [12].

Although several studies have focused on adolescents previously [13–32], there is no systematic analysis to summarize recent clinical trials and evaluate the efficacy and safety of TXA on AS operations. Therefore, in this article, we gathered previously published studies in order to carry out a systematic review and meta-analysis to explore the statistical significance of TXA treatment for hemostasis among AS surgery patients.

## Methods

### Search strategy and databases

English and Chinese electronic databases including Embase, PubMed, Web of Science, Cochrane, CNKI, and Wanfang database were searched to identify the studies published from the establishment of the study to August 2022. The reference lists of the resulting literature were also screened to find any initially omitted studies. The search method involved a combination of Mesh words and keywords. Take the PubMed database as an example, the searching strategy could be: ((tranexamic acid) OR (TxA) OR (AMCHA) OR (tranexamic acid) OR (AMCA) OR (Acidum tranexamicum)) AND ((posterior spine fusion) OR (lordosis) OR (kyphosis) OR (spinal curvatures) OR (“Scoliosis“[Mesh])) AND (adolescence). The integrity strategy is shown in Supplementary Fig. 1.

### Eligibility criteria and study quality

Literature selection was carried out according to the following inclusion criteria: (1) published randomized controlled trials (RCTs) and non-RCTs involving adolescents who underwent scoliosis surgery, including primary/secondary scoliosis, adolescent idiopathic scoliosis(AIS), etc.; (2) intervention including the administration of TXA and placebo or no treatment; (3) reported outcomes including one or more of the following criteria: the need for transfusion, interoperative blood loss, total blood loss, postoperative hemoglobin (Hb) level or the change in Hb level. Two reviewers (KC, LW) independently screened the literature and performed the quality assessment. Differences were resolved through discussion or referred to a senior researcher for a final decision. The RoB 2 tool was used for assessing the risk of bias of RCTs, which includes 5 domains: randomization process, deviations from intended interventions, missing outcome data, measurement of the outcome, and selection of the reported result. The Newcastle-Ottawa Scale (NOS) was used for the non-RCTs quality assessment, which evaluated 3 domains: selection, comparability, and outcome. Two researchers (QG, UM) independently screened the articles, extracted the data, and assessed the quality. Any disagreements were resolved by discussion with the involvement of a third person (ZZ, YS, HY). When viewing the articles, both researchers first viewed the abstract and keywords and then read the full text to decide whether they meet the inclusion criteria.

### Data extraction

All the selected literature was screened by researchers and the data were extracted simultaneously. The following data were extracted and recorded: (1) demographic information such as the number of patients, the ratio of male patients, and mean age; (2) preoperative Hb level and transfusion criteria; (3) dosage of TXA and subsequent infusion speed; (4) type of scoliosis and research type; (5) transfusion requirements (rate), intraoperative and total blood loss, postoperative Hb level, and the change in Hb level. All data were extracted and categorized into the TXA group and control group and summarized in Excel 2021.

### Outcome measures and statistical analysis

For the transfusion requirements (a dichotomous outcome), we calculated the RR with 95% confidence intervals (CIs) for presentation. Continuous outcomes (intraoperative blood loss, total blood loss, postoperative Hb level, and the change of Hb level) were expressed as the mean difference (MD) with 95% CIs. Stata, version 17.0 (Stata Corp., College Station, TX, USA) was used for the calculation of data. I^2^ and Q tests were used for the heterogeneity test. The fixed-effects model was adopted when there was no statistical evidence of heterogeneity (I^2^ < 50% or P-value > 0.1). Otherwise, a random-effect model was chosen. In the case of high heterogeneity, we used the sensitivity analysis method to reduce the articles that show differences in participants, surgical methods, and so on. The data of articles with high heterogeneity would be removed and subgroup analysis would be performed when the heterogeneity is still large. We assessed publication bias assessment by using Begg’s test and funnel plots (P value of > 0.05 means no bias).

We conducted the current systematic review and meta-analysis in accordance with the guidelines of the Cochrane Intervention System Review Manual, and the study was conducted according to the Preferred Reporting Items for Systematic Reviews and Meta-Analyses (PRISMA) declaration guidelines.

## Results

### Search results and quality assessment

**Fig. 1:**
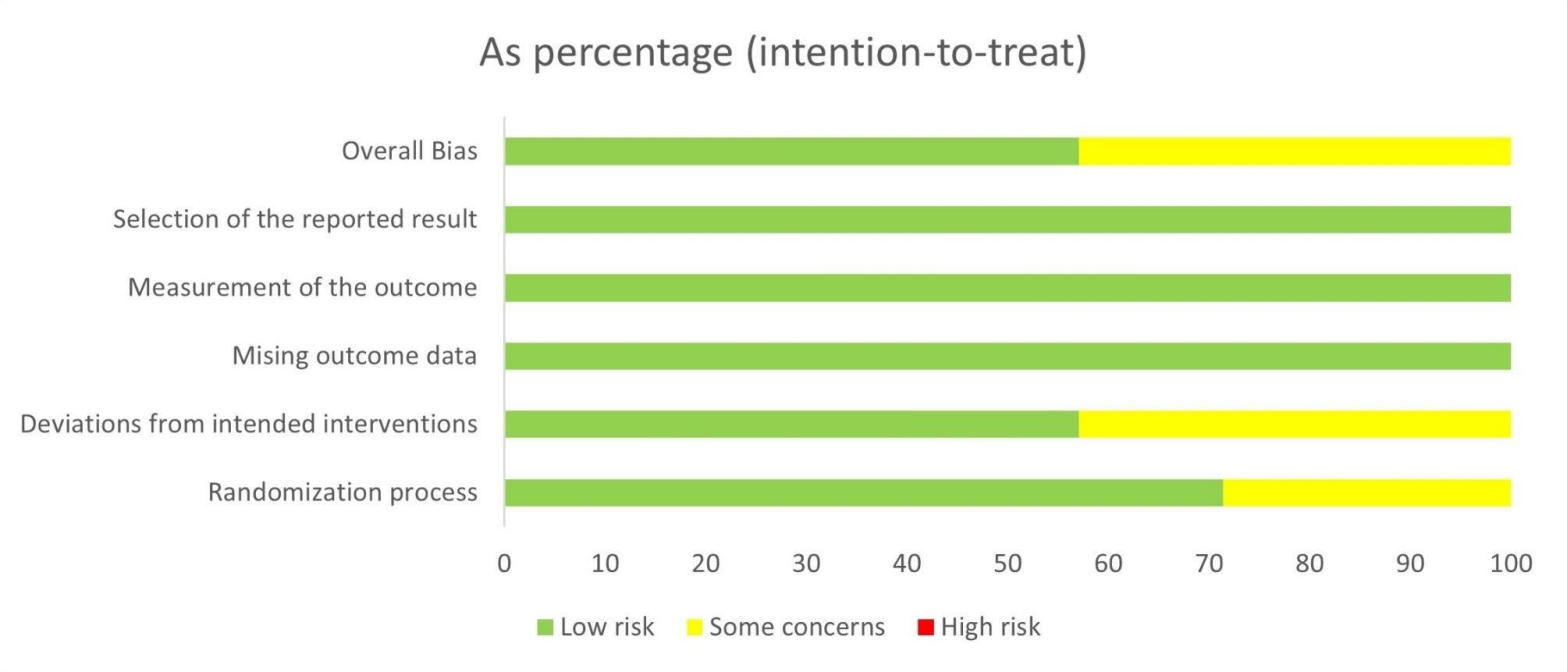
The bias assessment for RCTs. Deviations from intended interventions and the Randomization process have some concerns, and overall the RCTs have moderate concerns

**Fig. 2:**
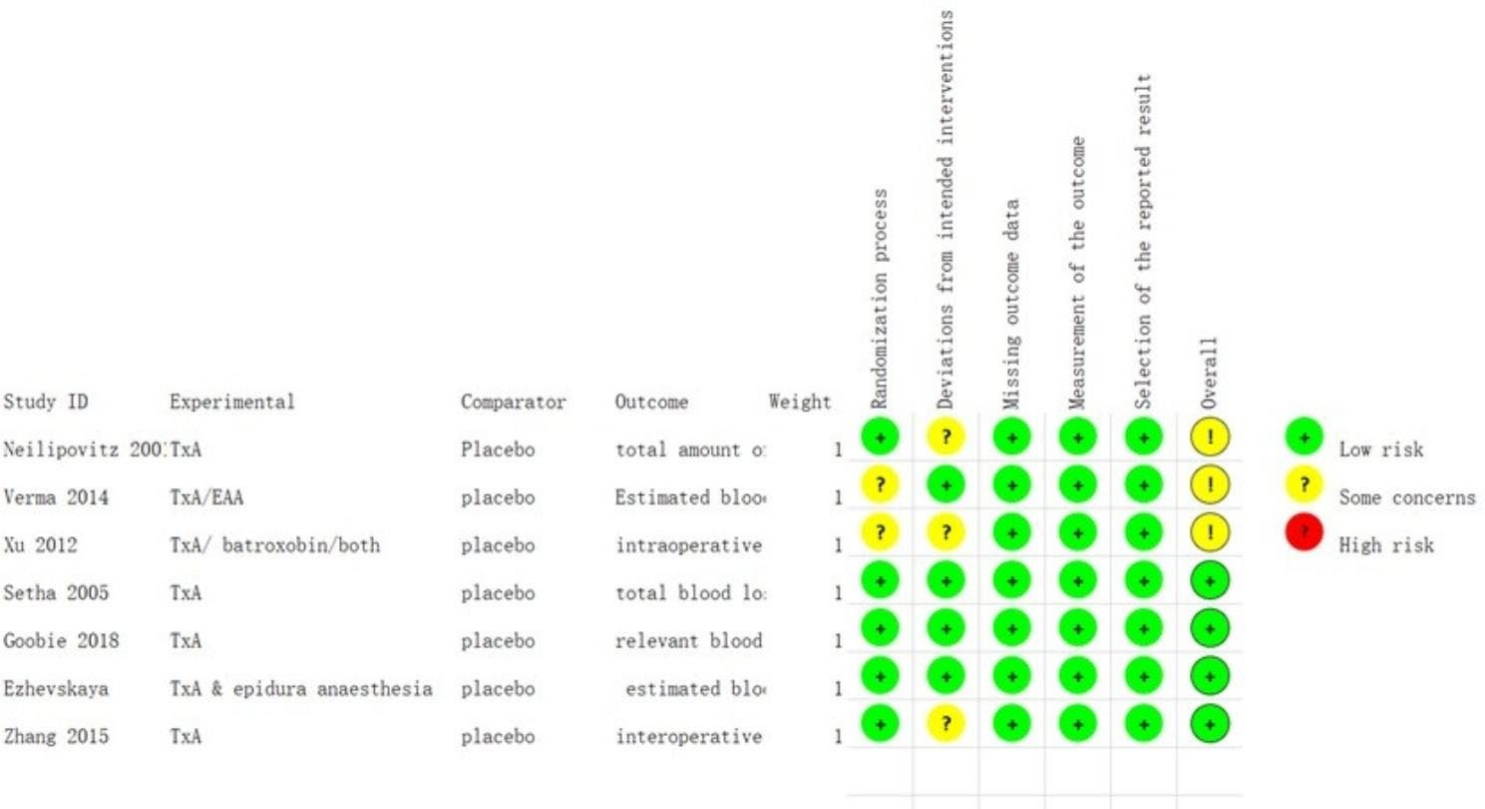
The bias assessment for RCTs. The specific evaluation of all articles shows that a total of 4 articles have concerns about their components

According to the search strategies, a total of 350 articles were identified: 50 from PubMed, 105 from Web of Science, 42 from Cochrane, 141 from Embase, five from CNKI, and seven from the Wan Fang database. After screening and assessing the quality, twenty articles ultimately met the inclusion criteria [13–32]. Among the selected items, there were 12 non-RCTs [13–15, 20–23, 25–27, 30, 31] and 8 RCTs [16–19, 24, 28, 29, 32]. Two articles were in Chinese [31, 32] and 18 were in English. All the articles were published after 2001. The average age of participants ranged from 13.7 to 21.6. The dose of TXA varied between studies, but the most typical dose was 100 mg/kg + 10 mg/kg/h. As for transfusion criteria, seven articles set the criteria at 7 g/dL, while three used 8 g/dL. Other articles had no or different criteria. The characteristics of each study were shown in Supplementary Table 1. The qualities of RCTs were presented in Figs. 1 and 2 (for RCTs) and Supplementary Table 2 (for non-RCTs). Most studies were of high quality and only 2 studies had concerns regarding the randomization process.

### Intraoperative blood loss

**Fig. 3:**
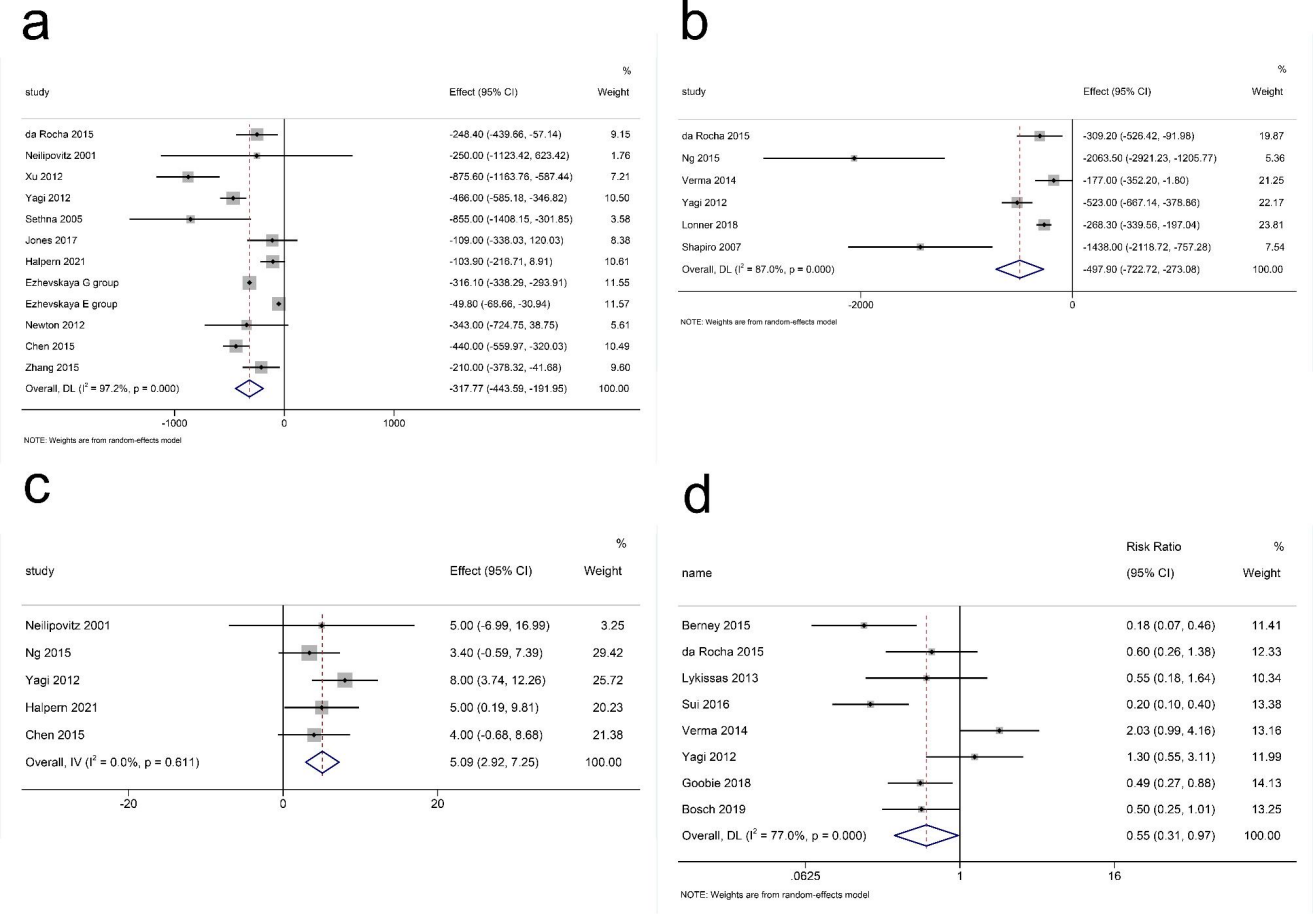
The overall assessment for different variables. **(a)** Interoperation Blood Loss; **(b)** Total Blood Loss; **(c)** Postoperative Hb Level; **(d)** Need for Transfusion. Axis Left: TXA group; Axis Right: Control group; Effect: Mean Difference; RR = Relative Risk; TXA = Tranexamic acid

**Fig. 4:**
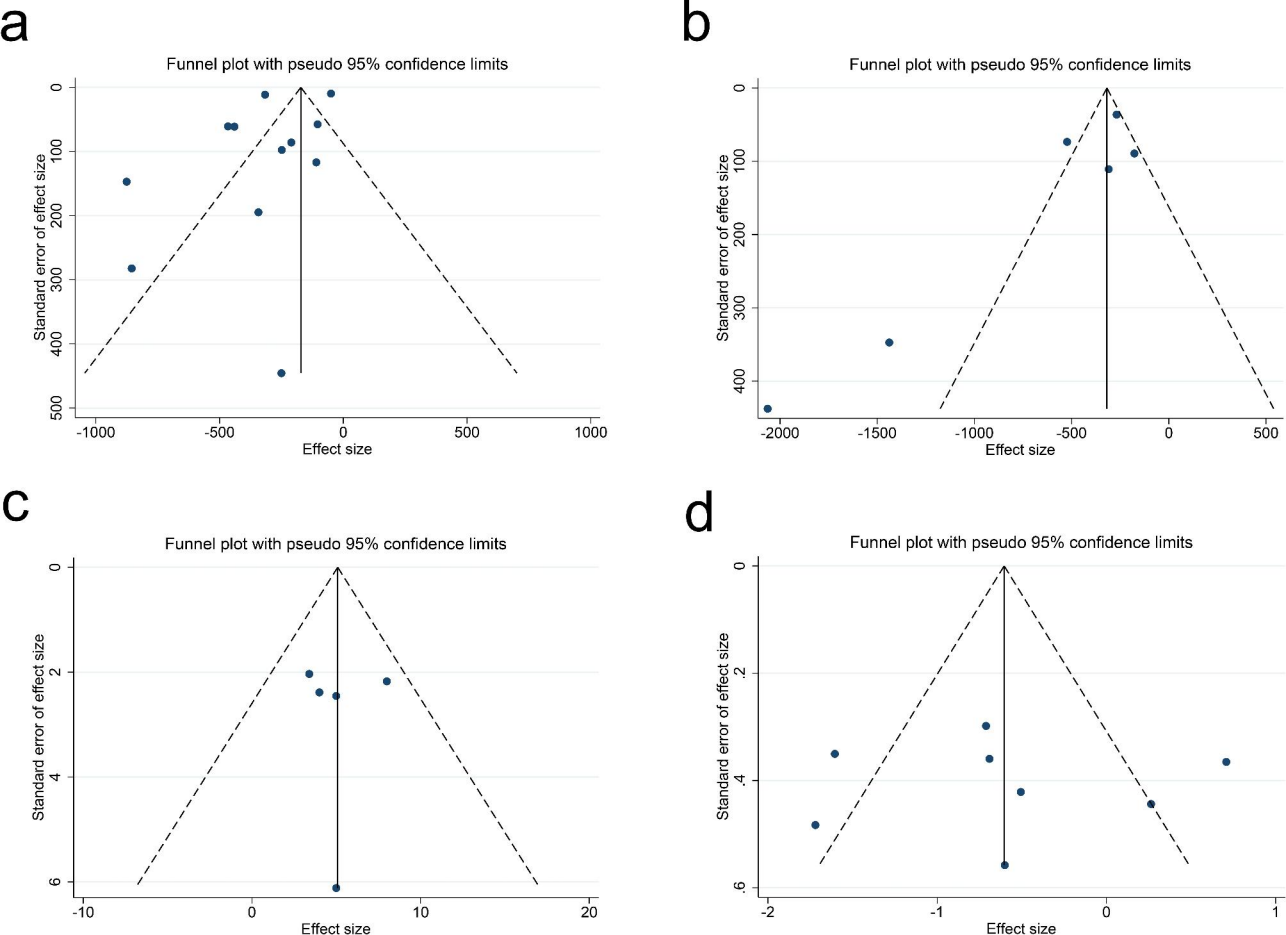
The funnel plot for different variables. **(a)** Interoperational Blood Loss; **(b)** Total Blood Loss; **(c)** Postoperative Hb level; **(d)** Need for Transfusion. Hb = hemoglobin

Twelve studies (795 participants) were included in the meta-analysis for intraoperative blood loss during surgery. Our results suggested that TXA reduced intraoperative blood loss in the patients (p < 0.001). There was a large heterogeneity among the studies (I^2^ = 91.8%, df = 11, p < 0.001)(Fig. 3a) with a funnel plot in Fig. 4a, so a random-effects model was adopted for our study.

### Total blood loss

Six articles (2027 patients) were included in the meta-analysis for total blood loss. The pooled result showed that the total blood loss of the TXA group was significantly lower than that of the control group (p < 0.001) with a large heterogeneity (I^2^ = 87.0%, df = 5, p < 0.001, Fig. 3b). There was no publication bias (p = 0.851), which was shown with a funnel plot in Fig. 4b.

### Postoperative and change in hb level

**Fig. 5:**
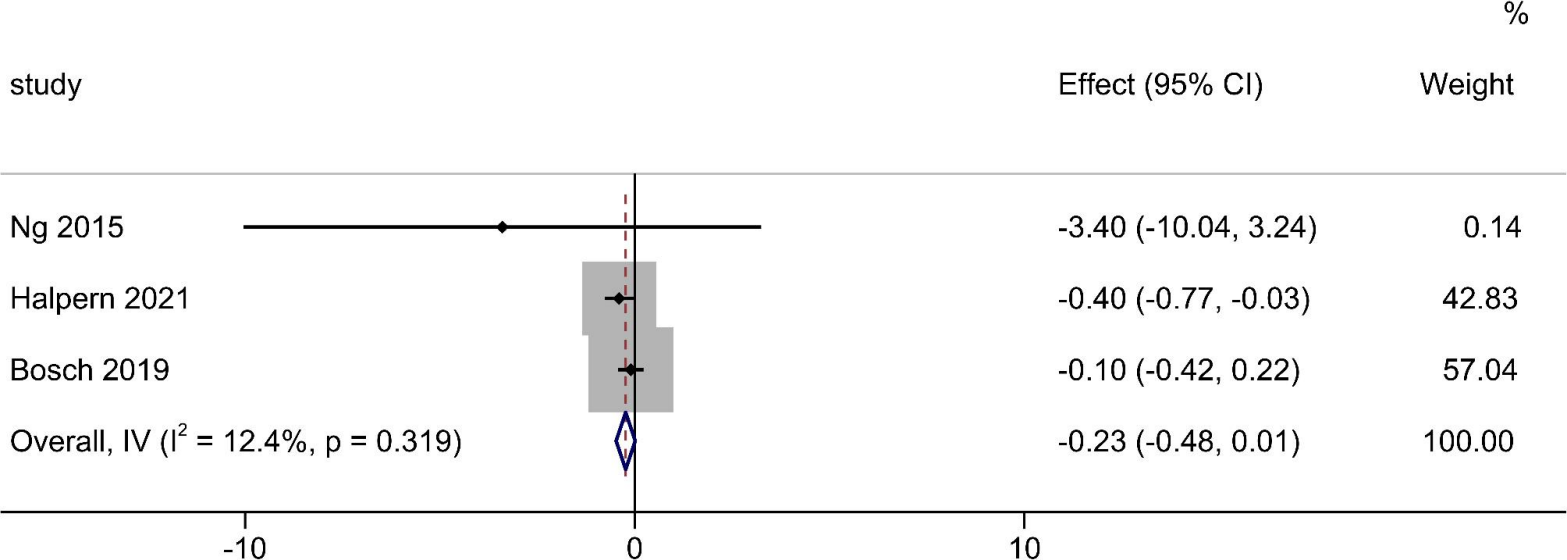
The overall assessment of the change in Hb level. Axis Left: TXA group; Axis Right: Control group; Effect: Mean Difference; TXA = Tranexamic acid; Hb = hemoglobin

**Fig. 6:**
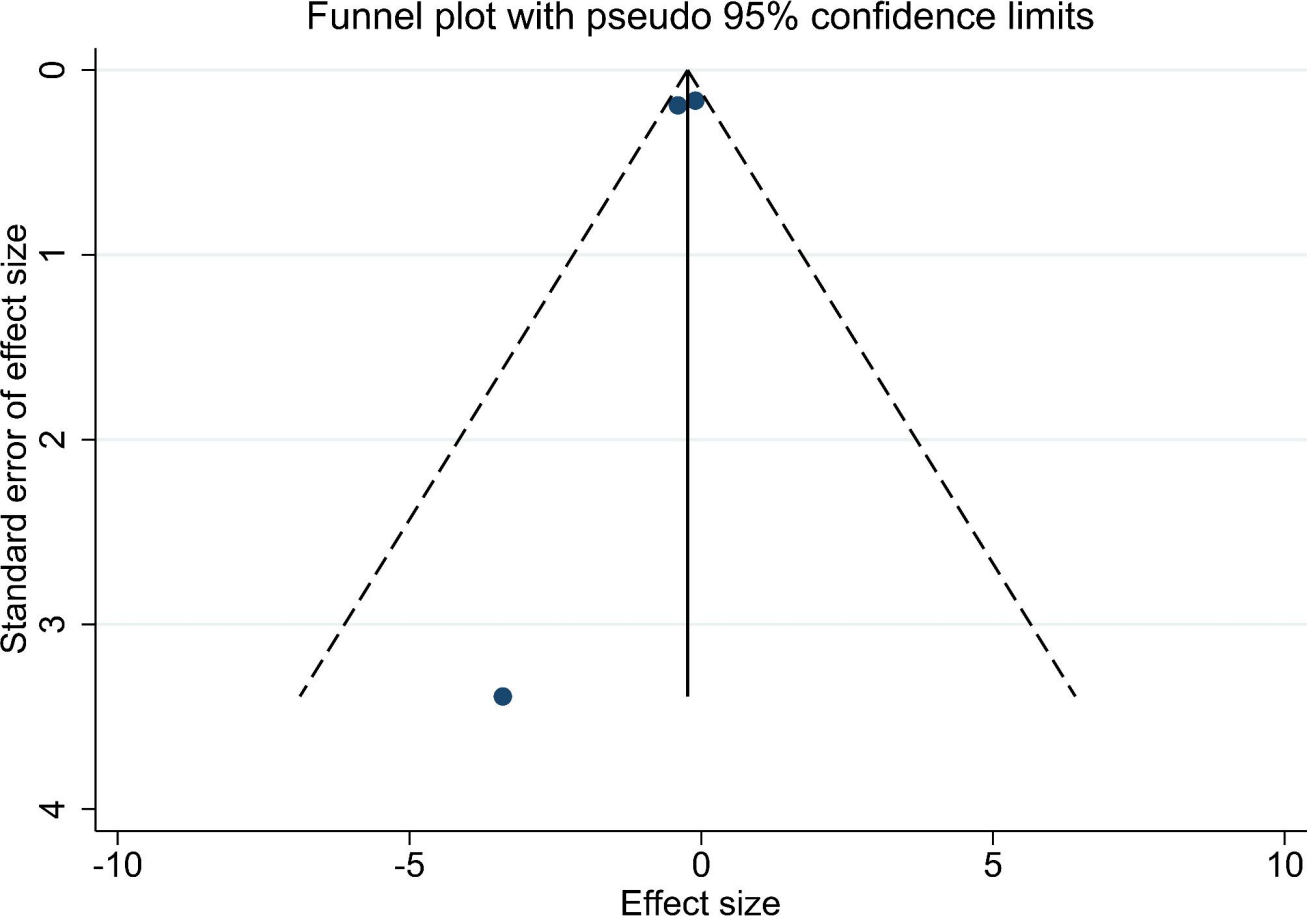
The funnel plot for the change in Hb level

Five articles (419 patients) were included in the meta-analysis for the postoperative Hb level. A small heterogeneity (I^2^ = 0.00%, df = 4, p = 0.611) (Fig. 3c) was shown with a significant outcome (p < 0.001). There is no publication bias for these articles (p = 0.327) (Fig. 4c). Three studies (268 patients) were included in the meta-analysis for the postoperative Hb level. There was a non-significant decrease in the TXA group (p = 0.093) with small heterogeneity (I^2^ = 12.4%, df = 2, p = 0.319) (Fig. 5) and no publication bias (p = 0.602, Fig. 6).

### Need for transfusion

A total of 8 studies (670 patients) had reported data on the patients that needed a transfusion after surgery. We used a random-effects model for the meta-analysis because of the high statistical heterogeneity (I^2^ = 77.0%, df = 7, p < 0.001) (Fig. 3d). The overall RR showed that there was a significant difference between the TXA and control group and that TXA could reduce the need for transfusion after surgery (RR = 0.547, 95%CI = 0.308–0.972, p = 0.04). There is no publication bias (p = 0.458) with a funnel plot in Fig. 4d.

### Sensitivity analysis and subgroup analysis

**Fig. 7:**
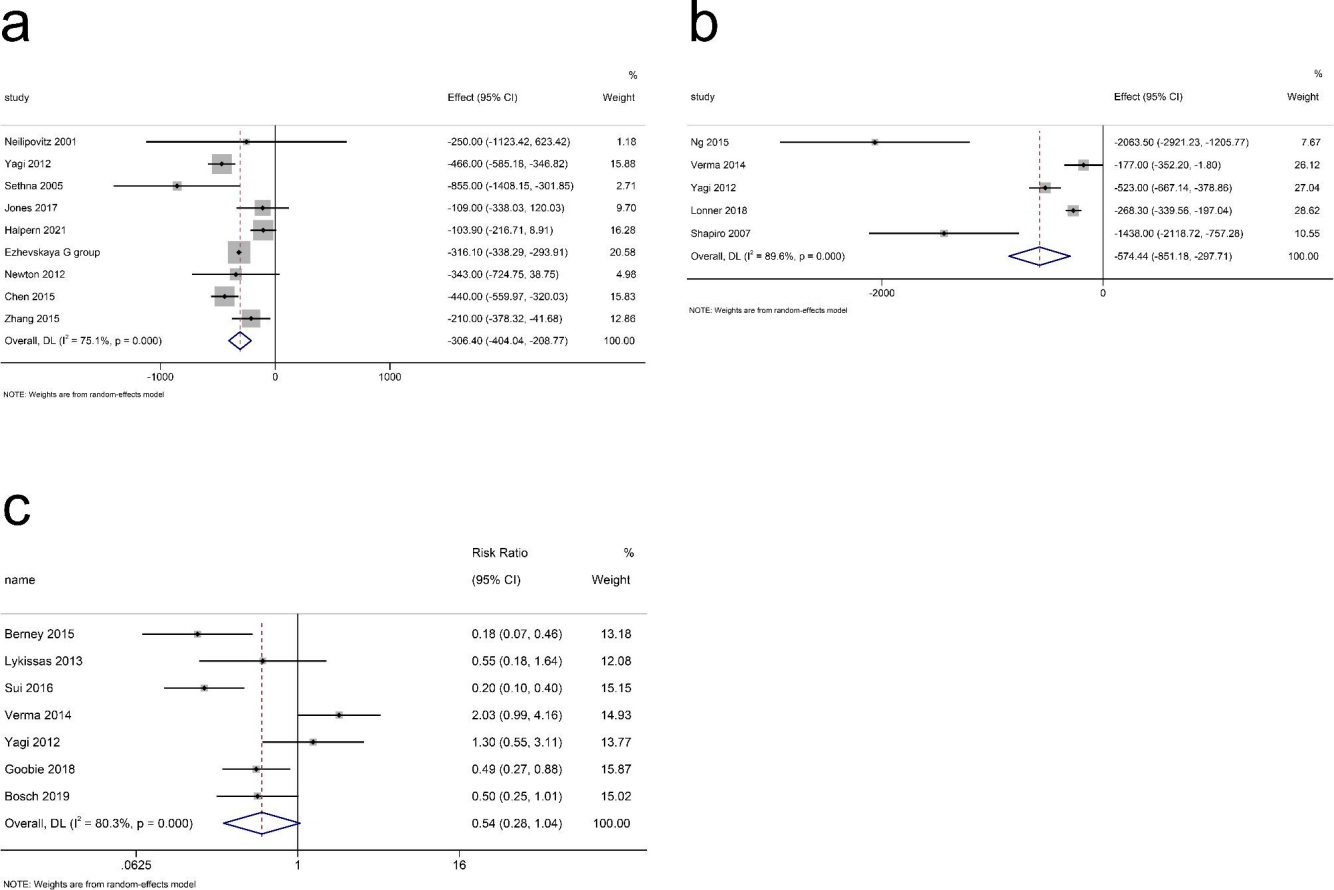
The pooled outcome for different variables after sensitivity analysis. **(a)** Interoperation Blood Loss; **(b)** Total Blood Loss; **(c)** The Need for Transfusion. Axis Left: TXA group; Axis Right: Control group; Effect: Mean Difference; RR = Relative Risk; TXA = Tranexamic acid

To address the issue of high heterogeneity in intraoperative blood loss, we conducted sensitivity analysis by reading the article one by one and eliminating the studies with the greatest heterogeneity impact. We requested the research subjects of the article and excluded studies with significant age differences and different anesthesia methods for further analysis. After we excluded 3 studies, the pooled outcome still had high heterogeneity (I^2^ = 75.1%, df = 8, p < 0.001) (Fig. 7a) with a significant outcome (p < 0.001) [15, 17, 29]. The Begg’s test indicates a small publication bias (p = 0.835). Subgroup analysis was performed and the outcome was shown in Table 1. We found that the cohort study (CS) subgroup had a large heterogeneity while the RCT group has a middle heterogeneity.

**Table 1:** Subgroup Analysis for Different Variables

Variables	**Studies**	p value	**Incidence**		
			**SMD(95%CI) OR RR(95%CI)**	Heterogeniety (I^2, p)	Model
Need for transfusion					
All	8	0.040	0.547 (0.308,0.972)	77.0%, 0.000	random
RCT	2	0.981	0.983 (0.245,3.950)	89.0%, 0.003	random
CS	6	0.007	0.440 (0.242,0.799)	67.2%, 0.009	random
Intraoperative blood loss					
All	12	0.000	-1.233 (-1.786,-0.681)	91.8%, 0.000	random
RCT	6	0.003	-1.800 (-3.001,-0.599)	95.0%, 0.000	random
CS	6	0.000	-0.775 (-1.211,-0.339)	79.7%, 0.000	random
Total blood loss					
All	6	0.000	-0.825 (-1.222,-0.428)	87.6%, 0.000	random
RCT	1	0.030	-0.488 (-0.929,-0.047)	\	\
CS	5	0.000	-0.901 (-1.393,-0.409)	84.6%, 0.000	random

As for total blood loss, after one studies [30] were eliminated, the pooled outcome (5 studies involving 1987 patients) indicated high heterogeneity (I^2^ = 89.6%, df = 4, p < 0.001) (Fig. 7b) and a significant decrease in the TXA group (p < 0.001) with potential publication bias (p = 0.05). Subgroup analysis was performed and the outcomes were shown in Table 1.

The same method was used for the five studies comprising 394 participants to analyze the transfusion requirement. After excluding one article [15], there was still large heterogeneity among the rest studies (I^2^ = 80.3%, df = 6, p < 0.001) (Fig. 7c). During the process, the pooled outcome didn’t change and the Begg’s test showed no publication bias (p = 0.453). Subgroup analysis was performed by research type and large heterogeneity was found in both subgroups (Table 1).

## Discussion

Thirteen CSs and seven RCTs were included in this study. Corresponding scoring tables were used for each included study to ensure the quality of the literature. Although no statistical difference in postoperative Hb content change, the application of TXA statistically and significantly reduced blood transfusion demand, intraoperative blood volume, and total blood loss.

Consistent with our results, many previous studies [13–35] have confirmed the effect of TXA on intraoperative hemostasis for AS patients in varying degrees. Berney et al. [13] reported that TXA use was related to fewer transfusion requirements and a mild decrease in Hb between preoperative and postoperative levels. Bosch et al. [14] found a lower transfusion rate rather than less blood loss in the TXA group. However, The TXA dosages are various among these studies. Goobie et al. [18] chose a higher TXA dosage (50 mg/kg load, 10 mg/kg/h infusion) to achieve a hemostatic effect. While in the CS of Halpern et al. [19], low TXA dosage (10 mg/kg load, 5 mg/kg/h infusion) could also significantly reduce the blood loss and transfusion requirements. TXA does have several adverse effects, such as gastrointestinal symptoms, allergy reaction [36], induction of seizure [37], transient hypotension [38], and even life-threatening thromboembolic events [39–42], which may be related to the dosage and usage of TXA. Therefore, it is necessary to review these studies extensively to identify the appropriate dose of TXA, contributing to optimizing surgical procedures and fusion criteria. However, in our meta-analysis, we did not find a significant difference in intraoperative blood loss among various TXA dosage groups (Supplementary Fig. 2). Therefore, a high-quality RCT is needed to discuss the optimal dosage and usage of TXA, determing the dose per weight per unit of time, administration method (intravenous injection, intravenous infusion, or subcutaneous injection), and dosing time.

The research included in our study was highly heterogeneous, which could impact the reliability of our findings. To ensure the validity of our results, we set the entry criteria as strictly as possible under the premise of sufficient literature included. Besides, we reassessed each article and deleted those with significant intra-group differences in participants’ age and surgical anesthesia mode out of our study cohort. After that, further analysis was conducted. Despite all these efforts, the final results still showed poor homogeneity. The subgroup analysis showed that a large amount of CS articles might be the reason for the high heterogeneity, particularly in the analysis of intraoperative blood loss. Additionally, different intervention timing and different patient visit time in CS studies were possible sources of heterogeneity because they were associated with different stages of the disease. However, due to the inconsistent evaluation method of RCT article results and the limited number of articles available for analysis, we can only temporarily accept the impact caused by CS.

In the analysis of blood transfusion demand, we found significant differences between the two intervention methods, which was an important supplement to previous research [1]. Consistent with previous studies, the overall heterogeneity was large. Since there may be slight differences in the standards of different researchers when conducting experiments, it is not surprising that the heterogeneity is substantial. Subsequently, we investigated the heterogeneity induced by each article through sensitivity analysis and found that one article had an extraordinary impact on the heterogeneity. After reading and reviewing the article, we proposed that the average age of the two groups may be an important source of heterogeneity. Tzortzopoulou et al. [43] also noted the important influence of antifibrotic solvents on heterogeneity. Therefore, the large heterogeneity of results in terms of blood transfusion demand was in expected. Furthermore, during sensitivity analysis, we found that the results of the study did not reverse, indicating that TXA alleviates the need for blood transfusion.

As another important comparison item, total blood loss showed statistical significance in this study, albeit with a high heterogeneity that required further attention. Firstly, we reviewed the number of fused spine levels in every study, and found it to have no difference between the TXA group and the control group (Supplementary Fig. 3). After that, we conducted a sensitivity analysis and excluded one article with high heterogeneity, which was also due to age differences. Although the heterogeneity of the articles keep high, the results did not change during the exclusion process and remained statistical significance.

Postoperative Hb level and change in Hb value are other two indicators that reflect plasma quality to a certain extent. We found that the TXA group had a small effect on preventing the decrease of Hb level, which was consistent with the outcome of Yuan et al. [5]. Though there was a significant difference in postoperation Hb level, the value (-0.23 g/l) is little bit lack of clinical meaning. Moreover, the change in Hb value had no significant statistical difference. There are several reasons to explain this unexpected result. On one hand, we were challenged by limited data reporting on postoperative Hb level and change in Hb value so the outcome needs further study to be verified. On the other hand, the Hb level is a dynamic index for anemia, which was affected by many factors such as sex, age, BMI, fluid balance, and blood transfusion [44]. In our opinion, it reflects the infusion strategy and transfusion policy better than the degree of blood loss when it comes to perioperative bleeding.

This study has inherent limitations. First, some indicators are highly heterogeneous in the process of analysis. We believe that the excessive heterogeneity is a result of our relatively broad inclusion criteria. The experimental conditions of many previous studies included were highly variable. Nevertheless, we chose broad inclusion criteria due to the limited number of existing articles. When the inclusion and exclusion criteria are set prospectively, many articles may be omitted, resulting in insufficient results. Establishing criteria in advance without knowing the information of the article could reduce author bias and lead to more accurate study conclusions.

We performed subgroup analysis and sensitivity analysis for certain studies. However, subgroup analysis alone was not adequate for some categories with only one study, which makes the subgroup analysis impossible. Therefore, we attempted to solve the problem through sensitivity analysis. Fortunately, sensitivity analysis identified some articles with high heterogeneity, and after exclusion post-review, the heterogeneity of pooled results did decrease. These findings explain the role of sensitivity analysis to a certain extent. However, we suggest that unknown factors affecting the heterogeneity might exist, which were not addressed in this article. Thus, we advise treating the results after sensitivity analysis with caution. To be noted, the results did not reverse in the process of sensitivity analysis, which increased the persuasiveness of our research results. Second, there were more CS articles. As mentioned previously, we believed that the intervention allocation of CSs is part of the reason for the higher heterogeneity. Although the impact cannot be avoided due to the limited number of experimental articles, this study further confirms the clinical significance of the hemostatic function of TXA, which was effective in reducing intraoperative bleeding by 306.40 ml and total bleeding by 779.24 ml.

## Conclusion

This study determined the significant impact of TXA treatment on reducing blood transfusion demand, intraoperative blood loss, and total blood loss. However, due to the high heterogeneity and limited data reporting in some indicators, caution is necessary when interpreting the results. In the future, we will conduct further studies with less heterogeneous and more controlled experimental research. Additionally, We also look forward to more high-quality studies reporting relevant results to improve our understanding of the usage of TXA in this population.

### Electronic supplementary material

Below is the link to the electronic supplementary material.

**Additional File 1**: Supplementary Table 1. Basic Information For Articles

**Additional File 2**: Supplementary Table 2. NOS for non-RCTs

**Additional File 3**: Supplementary Fig. 1. The PRISMA flow chart of retrieved studies

**Additional File 4**: Supplementary Fig. 2. The subgroup analysis by different TXA dosages for intraoperative blood loss. (a) Subgroup analysis by different loading dosages of TXA: 10 mg/kg, 15 mg/kg, 25 mg/kg, 50 mg/kg; (b) Subgroup analysis by different maintenance dosages of TXA: 1 mg/kg, 5 mg/kg, 10 mg/kg. TXA = Tranexamic acid; SD = Standard Difference; SMD = Standard Mean Difference; CI = Confidence Interval.

**Additional File 5**: Supplementary Fig. 3. The overall assessment of the number of fused spine levels. (a) The number of fused spine levels for studies mentioned intraoperative blood loss; (b) The number of fused spine levels for studies mentioned total blood loss. TXA = Tranexamic acid; SE = Standard Error; CI = Confidence Interval.

## Data Availability

Most of the original data are from the articles we referred to. The datasets used and/or analyzed during the current study are available from the corresponding author on reasonable request.

## List of abbreviations

AS: Adolescent scoliosis
AIS: Adolescent idiopathic scoliosis
CIs: Confidence intervals
CS: Cohort study
Hb: Hemoglobin
NOS: Newcastle-Ottawa Scale
PRISMA: Preferred Reporting Items for Systematic Reviews and Meta-Analyses
RCTs: Randomized controlled trials
RR: Relative risk
SMD: Standardized mean difference
TXA: Tranexamic acid
