# Supplementary figures and images for: Tranexamic acid can reduce blood loss in adolescent scoliosis surgery: a systematic review and meta-analysis
Source: BMC Musculoskelet Disord. 2023 Aug 29;24:686. doi: 10.1186/s12891-023-06811-1 (PMC10463947; doi:10.1186/s12891-023-06811-1)

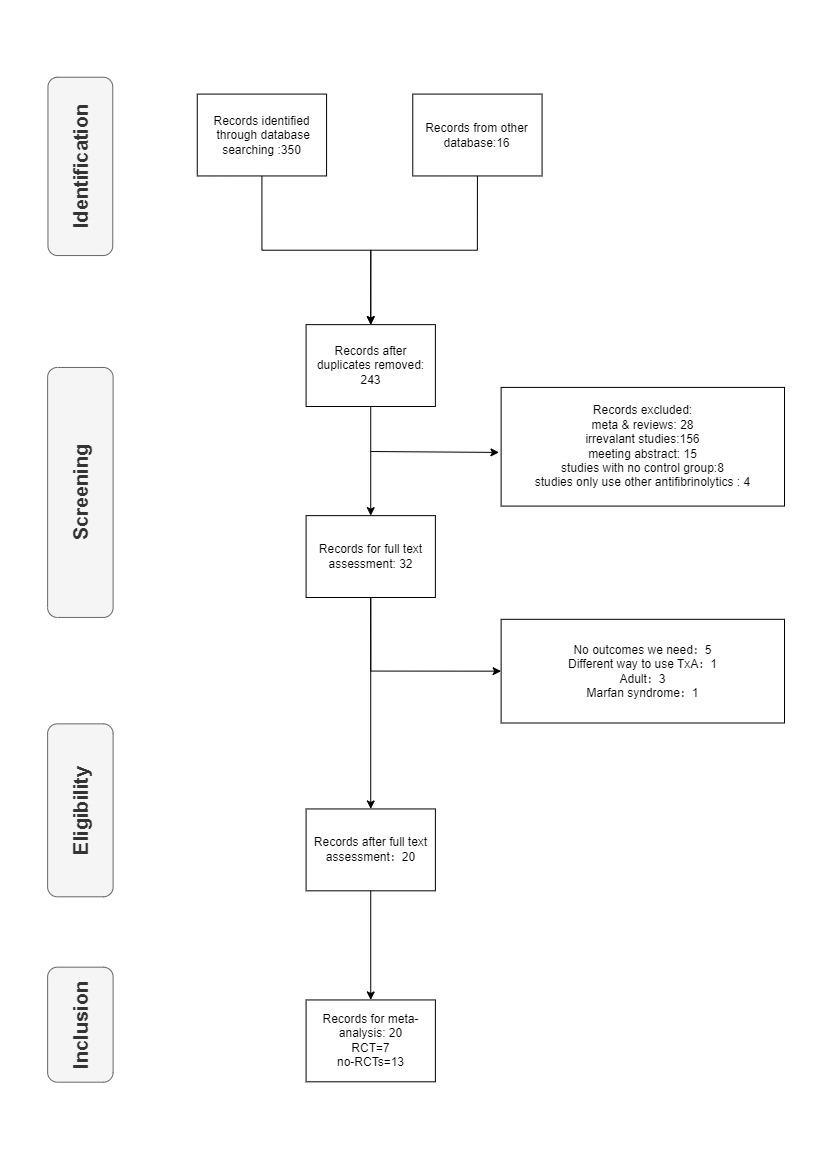


**Supplementary File Legends**

Supplementary Figure 1. Searching strategy for meta-analysis

Supplement: Supplementary file 3 — Additional File 3: Supplementary Fig. 1. The PRISMA flow chart of retrieved studies [file 12891_2023_6811_MOESM3_ESM.docx]

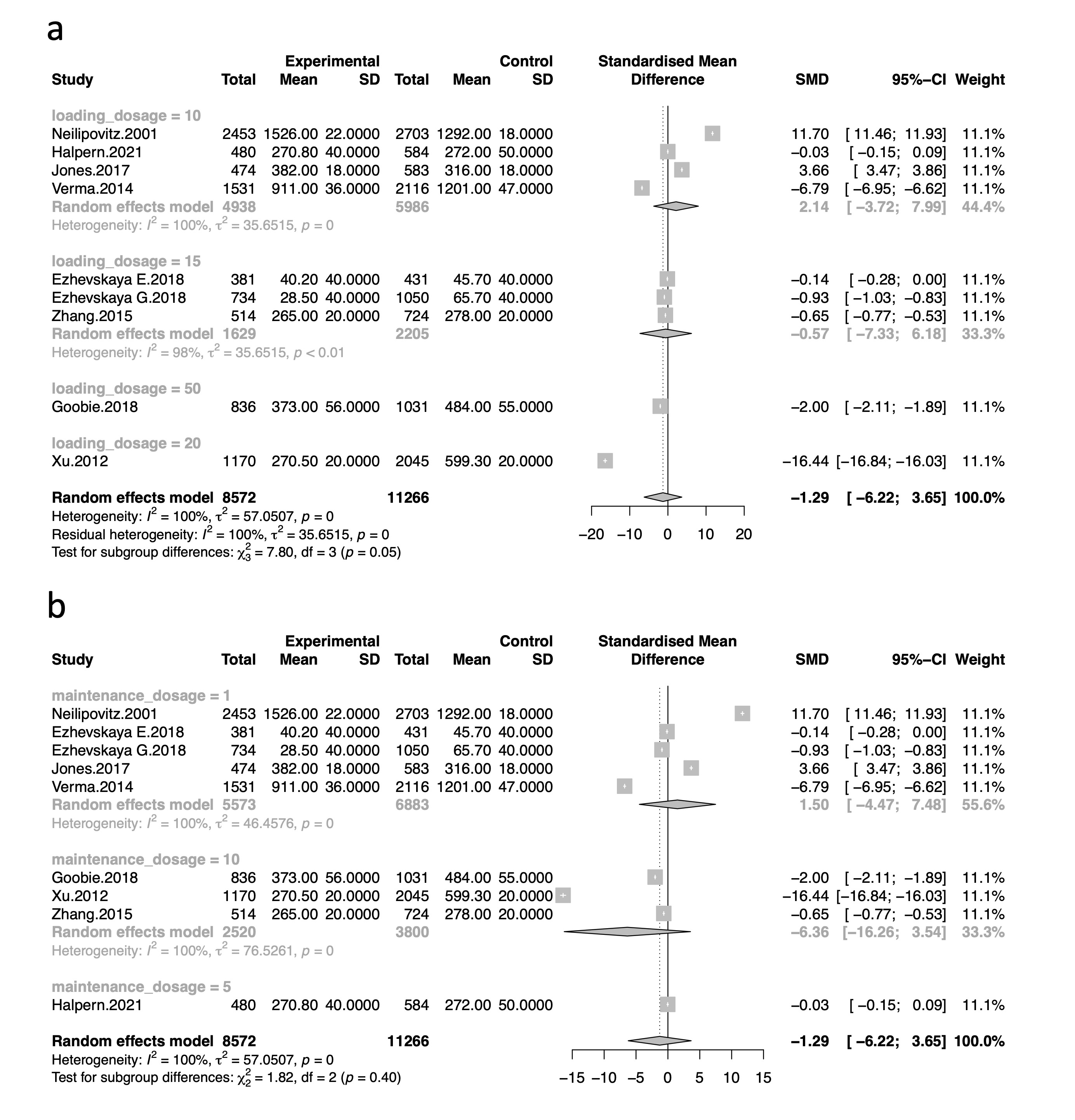

Supplement: Supplementary file 4 — Additional File 4: Supplementary Fig. 2. The subgroup analysis by different TXA dosages for intraoperative blood loss. (a) Subgroup analysis by different loading dosages of TXA: 10 mg/kg, 15 mg/kg, 25 mg/kg, 50 mg/kg; (b) Subgroup analysis by different maintenance dosages of TXA: 1 mg/kg, 5 mg/kg, 10 mg/kg. TXA = Tranexamic acid; SD = Standard Difference; SMD = Standard Mean Difference; CI = Confidence Interval. [file 12891_2023_6811_MOESM4_ESM.jpg]

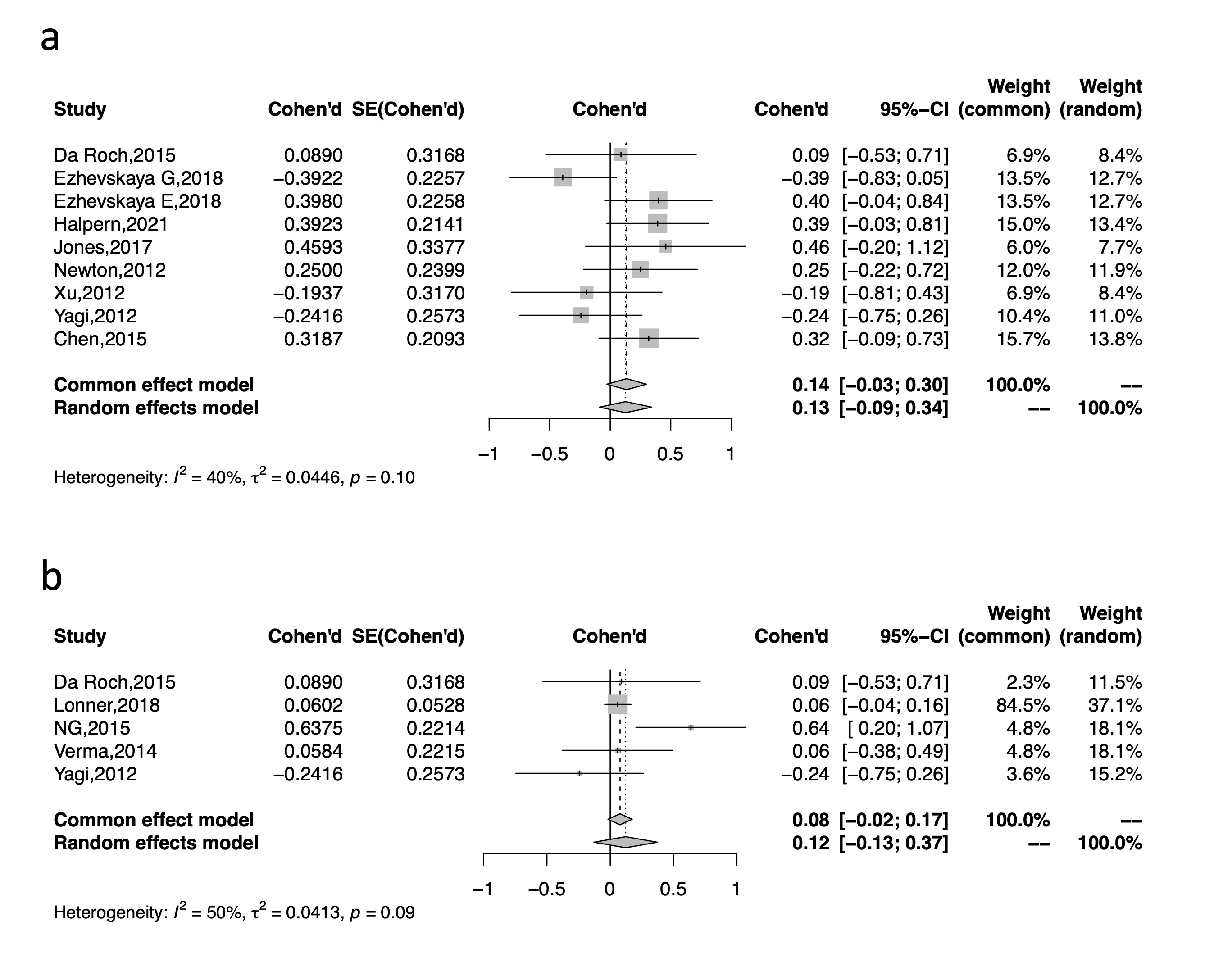

Supplement: Supplementary file 5 — Additional File 5: Supplementary Fig. 3. The overall assessment of the number of fused spine levels. (a) The number of fused spine levels for studies mentioned intraoperative blood loss; (b) The number of fused spine levels for studies mentioned total blood loss. TXA = Tranexamic acid; SE = Standard Error; CI = Confidence Interval. [file 12891_2023_6811_MOESM5_ESM.jpg]
